# An English Patient in a German Hospital

**Published:** 1923-05

**Authors:** E. Channing Matthews


### An English Patient in a German Hospital.

SIR,—" Patient ill on a Wednesday, and German doctor immediately called. An internal examination was carried out minus gloves or previous sterilisation. On Friday morning, however, an operation was necessary, and the patient was removed to an outside hospital. The heart was not sounded before the anæsthetic—ether—was given. The ether was administered by nurses entirely. After the operation, but later in the day, tea with no sugar or milk was given, and a little soup was taken by the patient before sleeping for the night. The next day there was Zweibach bread, margarine and tea with milk for breakfast, and for lunch, soup, minced meat and potatoes, and lastly stewed plums. Dinner—or supper, as it is called in Germany—concluded the day's menu. It consisted of pickled cucumber, German sausage, and raw ham served on chunks of bread and margarine. The patient expressed a desire for eggs, and, although a first-class patient in a private ward, she was told there were none to be had. Consequently, her meals were not at all appetising, especially as her tastes lay in the direction of simple foods, such as fresh butter and milk, which were absolutely unobtainable, excepting from an outside source. In the morning only the patient was washed with cold water (merely hands and face, however), but there was no soap used. The windows were practically always closed, and a German heat radiator was on the whole time. The bed was never fully 'made,' and so on—but despite all these extraordinary happenings, the patient still lives!"

E. Channing Matthews, F.R.M.S., M.I.H.

